# Surgical techniques and outcome assessment of a novel vascularized orthotopic rodent whole eye transplantation model

**DOI:** 10.1371/journal.pone.0311392

**Published:** 2025-05-23

**Authors:** Yang Li, Chiaki Komatsu, Lin He, Maxine R. Miller, Jila Noori, Yolandi van der Merwe, Leon C. Ho, Ian A. Rosner, Joshua M. Barnett, Kayvon Jabbari, Gadi Wollstein, Richard A. Bilonick, Valeria L. N. Fu, Mario G. Solari, An-Jey A. Su, Kevin C. Chan, Joel S. Schuman, Kia M. Washington

**Affiliations:** 1 Department of Plastic and Reconstructive Surgery, University of Pittsburgh School of Medicine, Pittsburgh, Pennsylvania, United States of America; 2 Department of Plastic and Reconstructive Surgery, Xijing Hospital, Fourth Military Medical University, Xi’an, Puerto Rico China; 3 Department of Plastic, Aesthetic, and Craniofacial Surgery, The First Affiliated Hospital of Xi’an Jiaotong University, Xi’an, Puerto Rico China; 4 Department of Ophthalmology, Eye and Ear Institute, Ophthalmology and Visual Science Research Center, University of Pittsburgh School of Medicine, Pittsburgh, Pennsylvania, United States of America; 5 Bascom Palmer Eye Institute, University of Miami, Miami, Florida, United States of America; 6 Department of Bioengineering, Swanson School of Engineering, University of Pittsburgh, Pittsburgh, Pennsylvania, United States of America; 7 McGowan Institute for Regenerative Medicine, University of Pittsburgh, Pittsburgh, Pennsylvania, United States of America; 8 Icahn School of Medicine at Mount Sinai, New York, New York, United States of America; 9 Department of Surgery, University of Colorado Anschutz Medical Campus, Aurora, Colorado, United States of America; 10 Department of Ophthalmology, New York University School of Medicine, New York, New York, United States of America; 11 Department of Biostatistics, University of Pittsburgh Graduate School of Public Health, Pittsburgh, Pennsylvania, United States of America; 12 Department of Radiology, New York University School of Medicine, New York, New York, United States of America; 13 V.A. Pittsburgh Healthcare System, Pittsburgh, Pennsylvania, United States of America; 14 Department of Orthopedic Surgery, University of Pittsburgh School of Medicine, Pittsburgh, Pennsylvania, United States of America; University of Iowa, UNITED STATES OF AMERICA

## Abstract

Currently there are no surgical solutions to restore vision in the irreversibly blind. Whole eye transplantation (WET), is an appealing surgical approach for restoration, replacement, and reconstruction of nonfunctioning eyes. Development of a reliable animal model to test the integrity and functionality of the transplanted eye is an essential step towards clinical whole eye transplantation. This study presents a feasible vascularized orthotopic eye transplantation preclinical rat model to study the structural and functional outcomes of whole eye transplantation. Syngeneic orthotopic transplants were performed in rats, involving anastomoses between carotid arteries, external jugular veins, and optic nerve coaptations of donors and recipients. The transplanted and recipient native eyes were assessed by ocular exam under anesthesia, optical coherence tomography (OCT), histology, magnetic resonance imaging and electroretinography. A 100% surgical survival rate of recipients with maintained long-term health demonstrated this to be a reliable and reproducible model. Assessment from clinical examination under anesthesia revealed that segments of native eyes appeared normal throughout the duration of the study, but transplanted eyes presented mild chemosis of the eye lids, mild ciliary flush of the conjunctiva, cornea neovascularization, mild engorgement of the vessels in the iris, and mild opacities in the lens in some animals. Most of these findings improved over time after transplantation. Doppler optical coherence tomography corroborated the presence of blood flow in transplanted retinas. There was no significant difference in measured IOP between native and transplanted eyes. Both histology and OCT scans demonstrated increased central corneal thickness and decreased total retinal thickness in transplanted eyes. Transplanted eyes exhibit minimal scotopic and photopic ERG responses. To date, no other vascularized orthotopic rodent WET transplantation models have been described in the literature. As functional visual return remains the ultimate goal, this model provides a foundation for future translational strategies and is ideal for testing immunomodulatory, neuroprotective, and neuroregenerative approaches either individually or in combination, as required for total human eye allotransplantation (THEA) to become a clinical reality.

## Introduction

Worldwide, an estimated 43.3 million people are blind, including a significant proportion who are irreversibly blind. For these cases, etiologies include malformation, infection, chronic disease, and trauma [1]. Visual disabilities significantly reduce quality of life, social interactions, and life expectancy which represents a global economic burden [2–4]. Currently there are no surgical solutions to restore vision in the irreversibly blind. However, over the past 20 years, vascularized composite allotransplantation (VCA) has emerged as a viable reconstructive option for treating carefully selected patients [5]. Different than solid organ transplantation (SOT), VCA includes the reconstructive transplantation of multiple tissues, including muscle, nerves, blood vessels, and skin from donor to recipient as a functional unit. VCA can restore form and function to patients with a severe injury or defect that are outside conventional reconstructive techniques. More than one hundred hand and face VCAs have been documented, with promising functional outcomes with graft survival that surpasses 95% when patients adhere to immunosuppressive regimes after antibody-based lymphocyte-depleting induction therapy and rehabilitative regimes [6,7]. Since the pioneering case performed in 2005, over forty facial allotransplantation (FT) procedures have been completed, producing remarkable functional outcomes [8–10]. Among all FTs cases, 54% included restoration of periorbital components. Among these FTs, 13% were blind [11,12] with at least three FT recipients presenting with total bilateral blindness. Insomuch, FT candidates with blindness seem to be good first candidates for VCA eye transplantation, due to their pre-existing immunosuppression requirement, along with the inherently close anatomic relation of ocular tissues with the face. Such factors might decrease the risk: benefit ratio in these patients compared with isolated eye transplantation.

The concept of eye transplantation dates back to 1885, when M. Chibret unsuccessfully transplanted a rabbit eye into a seventeen-year-old blind girl [13]. Over the next 100 years, reported attempts to restore functional vision in either mammalian models or humans by whole eye transplantation (WET) had all failed [14]. In 1977, the advisory council for the U.S. National Eye Institute (NEI) called for a “limited and thoughtful laboratory effort” in eye transplantation. The council concluded that “at present, any effort to transplant a mammalian eye is doomed to failure by the retinal ganglion cell axons’ inability to withstand cutting, by the difficulty of ensuring adequate circulation of blood to the transplanted eye, and lastly by immune rejection of foreign tissue” [15,16].

Given the success of VCA over the past two decades, VCA of the whole eye is an appealing surgical approach for restoration, replacement, and reconstruction of nonfunctioning eyes. Consideration of the recent advances in immunomodulation strategies, promising approaches to enhance nerve regeneration in the central nervous system (CNS), and an improved understanding of ischemia reperfusion injury (IRI), the possibility of future clinical WET has been reinvigorated [17,18]. For this to happen, eye transplantation still needs significant research and progress has been stymied by the lack of high throughput animal models to address the major barriers impeding translation to the clinic.

Development of reliable animal models is vital to optimize new and complex surgical procedures. Additionally, cadaveric studies and patient trials are prerequisites for any new clinical surgeries [19–27]. Historically, there has been great difficulty in establishing a consistent small animal model for conducting basic science research of eye transplantation [28–31]. Optimizing surgical revascularization techniques, immunomodulation, and neuroregenerative therapies all require using small-animal models that closely recapitulate human physiology and anatomy. Use of rodent models to investigate VCA outcomes is well established, dating to 1936 with the first reports of experimental VCA in rat by Schwind [32]. Compared with larger animal models, inbred rats are genetically identical, allowing control of many experimental variables [33]. Furthermore, the branching pattern of the arterial anatomy is comparable in rats and humans, with some notable differences. In the rat, the ophthalmic artery branches off the internal carotid and then travels along the optic nerve sheath. The rat central retinal artery branches from the ophthalmic artery and enters the globe to supply blood to the retina but does not penetrate the optic nerve sheath or head, as it does in humans. Therefore, experiments that investigate optic nerve damage and regeneration can include or exclude the effects of ischemia by separating the optic nerve from the central retinal artery. Previously, we established a rat hemifacial VCA model to study functional outcomes and cortical reintegration after FT [34,35]. The present study expands that model to include the entire ocular globe (whole eye), optic nerve, and its blood supply to yield a novel vascularized, orthotopic rodent eye transplant model for studying the structural and functional outcomes following WET.

## Results

### Eye transplant surgery and post-operative monitoring

**Fig 1:**
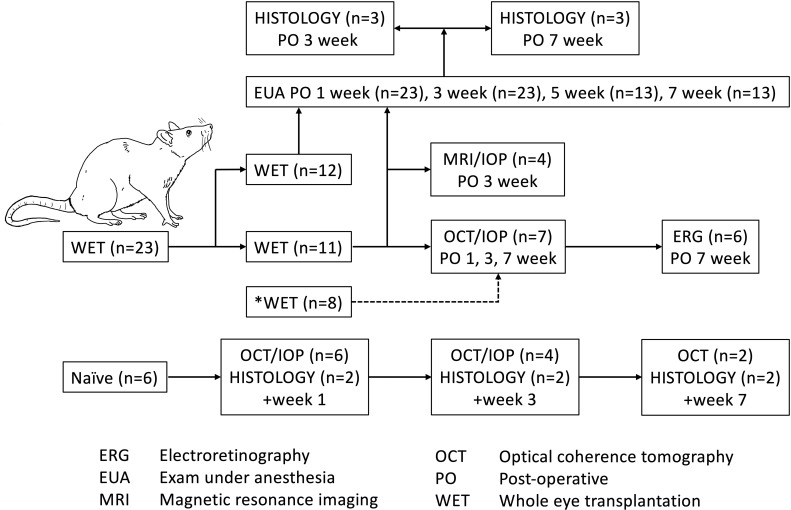
Experimental Design. Syngeneic whole eye transplantation was performed by using 23 pairs of age-matched male Lewis (RT1) rats to generate n = 4-6/group/time point for structural and functional assessment. Additional animals (dashed arrow) were used if ocular conditions prevented further analysis by optical coherence tomography and electroretinography. These WET animals were not used for ocular examination under anesthesia and histologic analyses.

**Fig 2:**
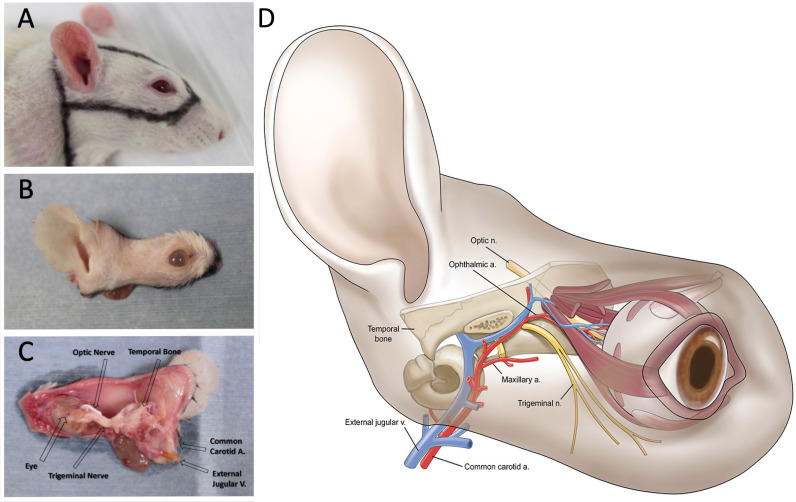
WET Donor Flap Preparation. A) An elliptical incision is made with a linear extension for exposure of the carotid artery and external jugular vein. B) Skin around the eye and auricle is included. C) Part of the temporal bone is left with the flap to protect the pterygopalatine and ophthalmic arteries and part of the trigeminal nerve posterior to the orbital content is preserved to protect the pterygopalatine artery running beneath its membrane. D) Schematic of donor flap.

**Fig 3:**
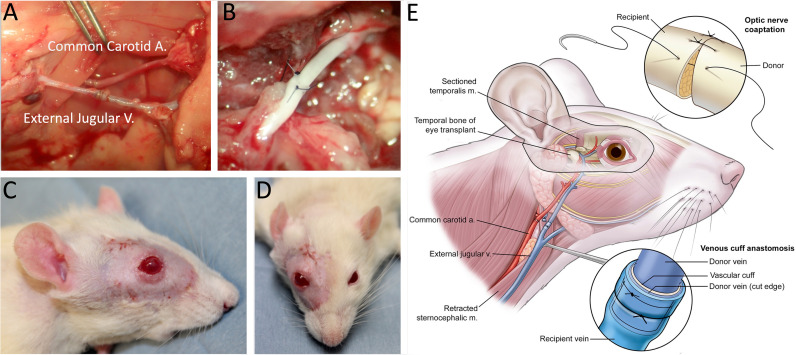
WET Recipient Procedure. A) Vascular anastomoses. B) Coaptation of the optic nerve. C) Lateral and D) frontal view of recipient at PO week 4. Note the comparable color in the eyes and ears between both sides, indicative of restoration of blood flow. E) Schematic of optic nerve coaptation, venous cuff anastomosis, and insetting of the flap. General topography of the nerve was maintained during coaptation of the nerve.

Syngeneic whole eye transplantation was performed using 23 pairs of age matched male Lewis (RT1) rats to generate n = 4-6/group/timepoint for downstream structural and functional assessment (Figs 1-3). Prior to surgery, eye examinations were performed to ensure donors and recipients were free of ocular pathology. Total surgical time for WET procedure was 358.8 ± 25.2 minutes (min) (mean±SD). Donor tissue recovery time was 136.8 ± 28.2 min (mean±SD). Recipient preparation time plus insetting the flap and globe was 3.16 ± 0.58 hours (mean±SD). Overall warm ischemia time was 2.22 ± 0.15 hours (mean±SD). All 23 WET rats survived the surgery (100%) and afterwards, the gross appearance of the flap and eyelid presented as healthy, pink, and well perfused (Figs 3C, 3D and SF1). Post-operatively, transplant recipients were monitored daily and qualitatively assessed for general health. After surgery, all animals presented normal behavior and health, appearing absent of distress. All animals were devoid of skin tenting and sunken eyes, which indicate dehydration. Transplant recipients immediately resumed normal food and water intake, allowing them to recover and maintain their pre-operative weights within post-operative (PO) week 1 (333.93 ± 24.52 g, mean±SD). Recipients were social, moved normally about their environment, resumed self-grooming and had normal breathing and posture.

### Ocular examination under anesthesia (EUA)

**Fig 4:**
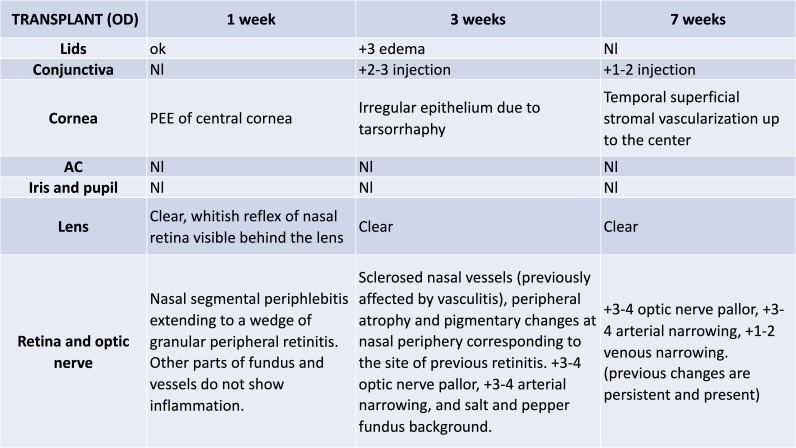
Representative example of EUA by clinically trained ophthalmologist. Notes reflect EUA for transplanted eye of a single recipient animal examined at 1, 3, and 7 weeks PO following WET. Fundoscopic examination evaluated viability and structural integrity. Slit lamp examination was used to assess the eyelids, conjunctiva, cornea, anterior chamber, iris, lens, and fundus. All rats examined under slit lamp had a vascularly intact graft with a patent vascular network. For the animal in Figure 4, there was mild chemosis and hemorrhage in the conjunctiva, mild perilimbal injections, mostly transparent cornea with some degree of peripheral neovascularization, mild-to-moderate cataracts, and red reflex elicited for all fundus exams. These ocular findings improved or remained the same during the follow-up period (see ST1). PEE = Punctate epithelial erosions. Nl = Normal.

The external, anterior, and posterior segments of globes were examined by a clinically trained ophthalmologist using slit lamp, fundoscopy and ophthalmoscopy (assessment included the eyelids, conjunctiva, cornea, anterior chamber, iris, lens, vitreous, retina, vessels, periphery, and optic nerve). EUA was performed bilaterally on naïve donor and recipient eyes prior to surgical procedures. Only animals in which all segments of the naïve eye appeared normal were included for this study. After WET, EUA of recipient native, contralateral (OS) and transplanted eyes (OD) was performed at PO weeks 1 (n = 23), 3 (n = 23), 5 (n = 13), and 7 (n = 13). Findings were scored as: 0 normal; 1 + mild; 2 + moderate; 3 + severe; 4 + very severe. Fig 4 is a representative example of longitudinal clinical findings recorded by the ophthalmologist for a single WET animal. All WET recipients (23/23) had intact vasculature and red reflex for both native and transplanted eyes at PO week 1.

All segments of native eyes were largely normal throughout the duration of the study, with the exception of one eye (1/23) presenting mild corneal edema with mild peripheral neovascularization, and 4/23 eyes with mild epithelial defects at PO week 1, all of which resolved by PO week 3. Mild corneal defects were found at PO week 7 in 2/13 eyes. Of these two eyes, one (1/13) also had mild neovascularization of the cornea at PO 7 week. Fundoscopic evaluation of the native retina and optic nerve head was normal for all animals at all of the examination sessions. No signs of inflammation were observed in either the vitreous or retina of native eyes at all exam timepoints.

At PO week 1, 4/23 transplanted eyes displayed mild chemosis of the eyelid, which resolved at 30 ± 4 days after transplantation. Peripheral corneal neovascularization was seen in 9/23 transplanted eyes, with initial heterogenous presentation: PO week 1 (n = 4/23 eyes), PO week 3 (n = 5/23 eyes), PO week 5 (n = 2/13 eyes), PO week 7 (n = 4/13 eyes) and this condition persisted in all cases with increased severity at subsequent exam timepoints. After WET, the lens remained clear in 21/23 transplanted eyes until the sacrifice endpoint. Mild-to-moderate cataracts were observed in two transplanted eyes (2/13) at PO 7 week. Of these two affected eyes, one also presented with a severe epithelial defect with moderate peripheral neovascularization in the cornea. In the second case, severe corneal edema obstructed the view of the posterior segment. The other 21 eyes were unobstructed, presenting a clear cornea and lens at each exam, but all the irises were dilated. At all exam time points, observable pupils were dilated and non-reactive for transplanted eyes. Anterior chamber inflammation with turbid view was detected in only one case at PO week 3 (1/23) and this condition persisted until the study endpoint. At PO week 1, 23/23 WET retinas were visible and attached, but as mentioned above, posterior segment exam was eventually limited in two transplanted eyes (2/13) due to a blocked view secondary to an opaque cornea at PO week 7. Of these transplanted eyes, 10/11 presented mild-to-severe peripheral ischemia at PO week 1. This resolved in all eyes except for 2 cases by PO week 3, which then persisted until the study endpoint. In all cases, the peripheral retina appeared to be less perfused than the posterior pole. Most optic disc and posterior pole regions of the retina appeared to be perfused (22/23) at PO week 1, although 10/23 were hypoperfused. Optic nerve heads appeared pink, healthy, and perfused in all observable cases, except for one eye with mild pallor at PO weeks 3 and 5, which increased to a total of two eyes at PO week 7. The vitreous humor was clear and free of inflammation in all observable animals throughout the course of the study.

### Optical coherence tomography (OCT) and Doppler OCT (ODT)

**Fig 5:**
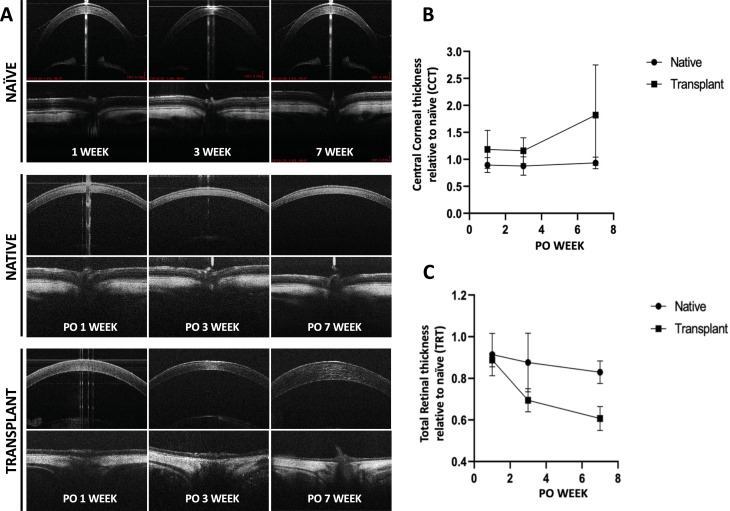
Optical Coherence Tomography (OCT) b-scans of the cornea and retina from A) age-matched naïve, native, and transplanted eyes. Representative images demonstrate gross maintenance of ocular structures in transplanted eyes from PO 1-7 weeks following WET. Measurements of SD-OCT scans for B) central corneal thickness (CCT) and C) total retinal thickness (TRT) are plotted for native and transplanted eyes relative to naïve measurements. Following WET, transplant eye CCT was thicker relative to native or naïve eyes, with TRT thinning of the transplanted retina that was particularly apparent within the anterior hyperreflective layer.

OCT imaging was performed serially on a subset group of recipients (n = 7) in native and transplanted eyes at PO weeks 1, 3, and 7 to evaluate the cornea, lens, retina, and optic nerve head (Fig 5A). For this group, warm ischemia time was 2.30 ± 0.28 hours (mean±SD). When possible, OCT scans acquired from these seven rats were used to measure total retinal thickness (TRT) and central corneal thickness (CCT) of native and transplanted eyes after WET. Average values were compared with TRT and CCT of age- and sex-matched naïve eyes (Fig 5B, 5C). Fig 5B shows the CCT and Fig 5C depicts the TRT at each timepoint relative to naïve eyes. Naïve CCT did not change significantly over the course of the study (PO week 1: 132.29 ± 10.79 µm, PO week 3: 140.89 ± 6.67 µm, PO week 7: 142.66 ± 1.52 µm, mean±SD), with a pooled average CCT of 139.40 ± 7.07 µm across all timepoints (pooled mean±SD, f: 2,27, ANOVA, *P* > 0.05). Naïve TRT was similarly stable over the course of the study (PO week 1: 204.68 ± 5.28 μm, PO week 3: 201.38 ± 3.39 μm, PO week 7: 205.12 ± 3.95 μm, mean±SD; *P* > 0.05). Naïve TRT and CCT were normal, measuring within the range of reported values from the literature [36].

Native CCT at PO week 1 was 168.22 ± 19.26 µm, and this mean value did not change significantly at subsequent timepoints, measuring 178.00 ± 20.60 µm at PO week 7 (*P* > 0.05; Fig 5B). Following WET, transplanted eye CCT was thicker than either native or naïve eyes at each timepoint, with a marked increase at PO week 7, measuring 342.34 ± 80.03 µm or approximately two-fold thicker relative to naïve CCT. However, due to variability in the measurements among transplanted eyes (likely due to eye condition, see below), this increase was just outside statistical significance (*P = *0.53; Fig 5B).

Following WET, transplanted eye TRT decreased significantly 7 weeks PO relative to naïve or native eyes. Native TRT after WET was stable, measuring: 185.80 ± 3.55 µm at PO week 1 and 177.40 ± 7.44 µm at PO week 7 (*P* > 0.05, Fig 5C). Transplanted eye TRT was thinner at PO week 3 compared with either naïve or native eye TRT (*P = *0.03) and continued to decrease, measuring approximately ~ 50% thinner than naïve TRT at PO week 7 (Fig 5C).

Evaluation of OCT scans found that in 5/7 native and 3/7 transplanted eyes, the anterior chamber had fibrinous reactions (ST1). In the transplanted eyes with anterior chamber fibrinous reaction, corneas appeared thicker with epithelial irregularity. Corneal epithelial thickening and irregularity without fibrinous reaction was found in one of the transplanted eyes and one native eye. Along with corneal thickening, neovascularization was present in transplanted eyes, increased inner layer hyperreflectivity was found in 2/4 of transplanted eyes, and lenticular opacification was observed in transplanted eyes at PO week 3 (SF2). Due to formation of the lenticular opacity, retinal imaging was only possible in 4/7 animals after PO week 1. Of these 4 transplanted eyes, retinal OCT had low signal strength in 3/4 retinal OCTs, which made measurement of retinal layers difficult (SF2C). ODT of the retina was performed on this cohort and confirmed restoration of arterial and venous flow in all the retinas and optic nerves of the transplanted eyes (4/4) where OCT visualization of the posterior pole was possible (SF2C).

### Histological staining and evaluation of WET cornea and retina

**Fig 6:**
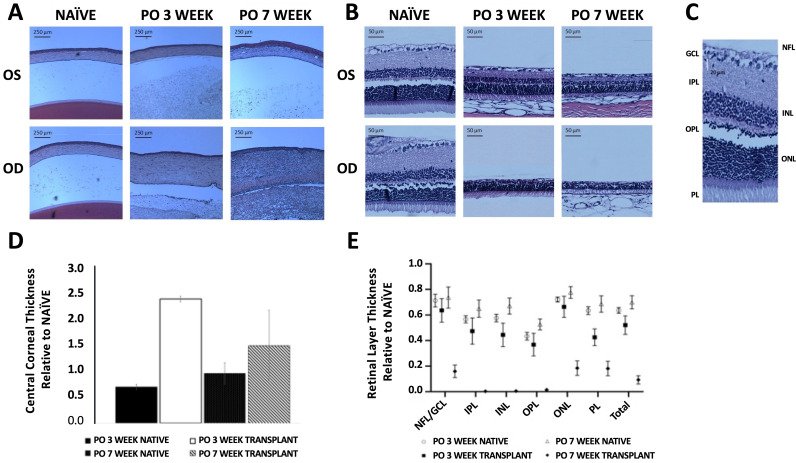
Histological changes in retina and cornea after WET. Representative examples of hematoxylin and eosin (H&E) stained A) corneas of naïve and recipient native (OS) and transplanted (OD) eyes at PO weeks 3 and 7 show corneal thickening in transplanted eyes compared to naïve eyes. B) retinas from naïve and recipient native (OS) and transplanted (OD) eyes at PO weeks 3 and 7 show retinal thinning in both native and transplanted eyes compared to naïve eyes. C) Representative image of retinal layers with labels. Histological samples were measured to assess the average D) central corneal thickness (CCT) and E) total retinal thickness (TRT) for native and transplanted eyes after WET and was plotted relative to values from age and sex-matched naïve eyes. Additionally, measurements from identified retinal layers including: the retinal Nerve Fiber Layer (NFL), Ganglion Cell Layer (GCL or NFL-GCL as the two were difficult to distinguish), Inner Plexiform Layer (IPL), Inner Nuclear Layer (INL), Outer Plexiform Layer (OPL), Outer Nuclear Layer (ONL) and Photoreceptor Layer (PL) plotted relative to naïve values at PO weeks 3 and 7 for native and transplanted eyes.

After WET, recipients were sacrificed, and the native and transplanted eyes were collected at PO weeks 3 and 7 to evaluate the condition of the cornea and the retina following WET. Additionally, sex and age-matched naïve eye pairs (n = 3; OD & OS) were collected for comparison. Fixed, enucleated eyes were sectioned and then stained by hematoxylin and eosin (H&E) for morphometric analysis (Fig 6A, 6B). The warm ischemia time for the WET cohort used for histological analysis at PO weeks 3 and 7 was 120.00 ± 14.24 min (mean ±  SD). Naïve CCT did not vary significantly throughout the study, measuring 129.24 ± 10.96 µm and 139.82 ± 15.13 µm at PO 3 and 7 weeks respectively (mean ±  SD; two-tailed paired t-tests, *P = *0.29).

For WET recipients, the native eye CCT measured thinner relative to naïve CCT at PO week 3 (two-tailed paired t-tests, *P = *0.03). This temporary thinning did not persist; the CCT of native eyes at PO week 7 measured insignificantly different from naïve CCT (135.16 ± 34.09 µm, mean±SD, *P = *0.11; Fig 6D). Transplanted eye CCT initially increased nearly ~ 2.5-fold relative to naïve corneas at PO week 3 (two-tailed paired t-tests, *P* = 0.001). At PO week 7, transplanted eye CCT remained thicker relative to naïve, although the transplant CCT decreased approximately ~ 40% from PO week 3, measuring 1.76-fold thicker relative to naïve eyes, with an average value of 247.18 ± 55.47 µm (mean ±  SD, two-tailed paired t-tests, *P = *0.03; Fig 6D).

TRT for naïve animals was insignificantly different when recorded at PO 3 and 7 weeks, measuring 196.71 ± 20.94 µm at PO week 7 (mean ±  SD, P > 0.05*)*. At PO week 3, the native TRT measured 118.60 ± 30.70 µm or 39.7% thinner relative to naïve retinas. No further reduction in native TRT occurred after PO week 3, averaging 108.58 ± 8.54 µm at PO week 7 (mean ±  SD, *P = *0.53; Fig 6E). On the other hand, the average transplanted eye TRT was thinner at PO week 3 (65.56 ± 8.58 µm) and continued to decrease, when measured at PO week 7 (17.51 ± 5.81 µm; two-tailed paired t-tests, *P = *0.03; Fig 6E). Transplanted eye TRT was 52.1% of naïve eye TRT at PO week 3, falling to 16.1% of naïve TRT at PO week 7 (two-tailed paired t-tests, *P < *0.001). For comparison, at PO week 7, transplanted eye TRT was 77% thinner relative to native TRT or approximately ~ 22% the native TRT (two-tailed paired t-tests, *P < *0.001).

### Retinal layers measurements

The following retinal layers were analyzed from histological samples of naïve, native and transplanted eyes: Retinal Nerve Fiber Layer-Ganglion Cell Layer (NFL-GCL), Inner Plexiform Layer (IPL), Inner Nuclear Layer (INL), Outer Plexiform Layer (OPL). At both PO 3 and 7 weeks, all retinal layers for both native and transplanted eyes displayed thinning relative to naïve eyes. For the transplanted eye, the reduction in layer thickness at PO week 3 further decreased for all layers measured at PO week 7. Among the transplanted eye retinal layers, the IPL, OPL and INL were most significantly affected, with values approaching zero µm by PO week 7 (Fig 6E). In transplanted eyes, the NFL-GCL and PL layer thickness decreased to less than 20% of the corresponding naïve values at PO week 7 (two-tailed paired t-tests, *P < *0.001). At PO week 3, the mean NFL-GCL thickness of naïve, native, and transplanted retinas was 19.60 ± 4.19 µm, 14.11 ± 4.30 µm, and 7.19 ± 5.89 µm, respectively (mean±SD). Thus, at PO week 3, native and transplant NFL-GCL thickness measured 28% and 63% thinner relative to naïve NFL-GCL, respectively. At PO week 7, the mean NFL-GCL thickness of naïve, native, and transplanted retinas was 20.70 ± 2.17 µm, 12.23 ± 5.19 µm, and 3.230 ± 1.31 µm (mean±SD), respectively.

### Tonometry Intraocular Pressure (IOP)

IOP was measured serially at PO 1, 3 and 7 weeks for both native and transplanted eyes of the subset of WET animals used in the OCT cohort (n = 7). Recorded IOP was compared with IOP of age-matched naïve eyes (n = 6, 4, or 2/timepoint, respectively). There was no significant difference in measured IOP between native and transplanted eyes at all timepoints (9.33 ± 0.57 *vs* 12.30 ± 2.08 mmHg, 9.75 ± 0.50 *vs* 13.80 ± 4.03 mmHg, 9.67 ± 1.37 *vs* 12.50 ± 3.08 mmHg, respectively). IOP was not significantly different at all timepoints for transplanted eyes relative to naïve eyes. Interestingly, native eye IOP measured significantly lower than naïve controls at PO 1, 3, and 7 weeks (Mann-Whitney test, *P* = 0.0004, < 0.0001, < 0.0001 respectively; SF3). In addition to IOP measurement of the OCT cohort, another group was assessed for IOP prior to MRI at PO week 3. IOP was measured bilaterally before magnetic resonance imaging (MRI) experiments (see MRI section in Results). In brief, native and transplanted eyes had normal IOP values.

### Gadolinium-enhanced magnetic resonance imaging (Gd-MRI), anatomical T2-Weighted MRI and diffusion tensor MRI (DTI)

**Fig 7:**
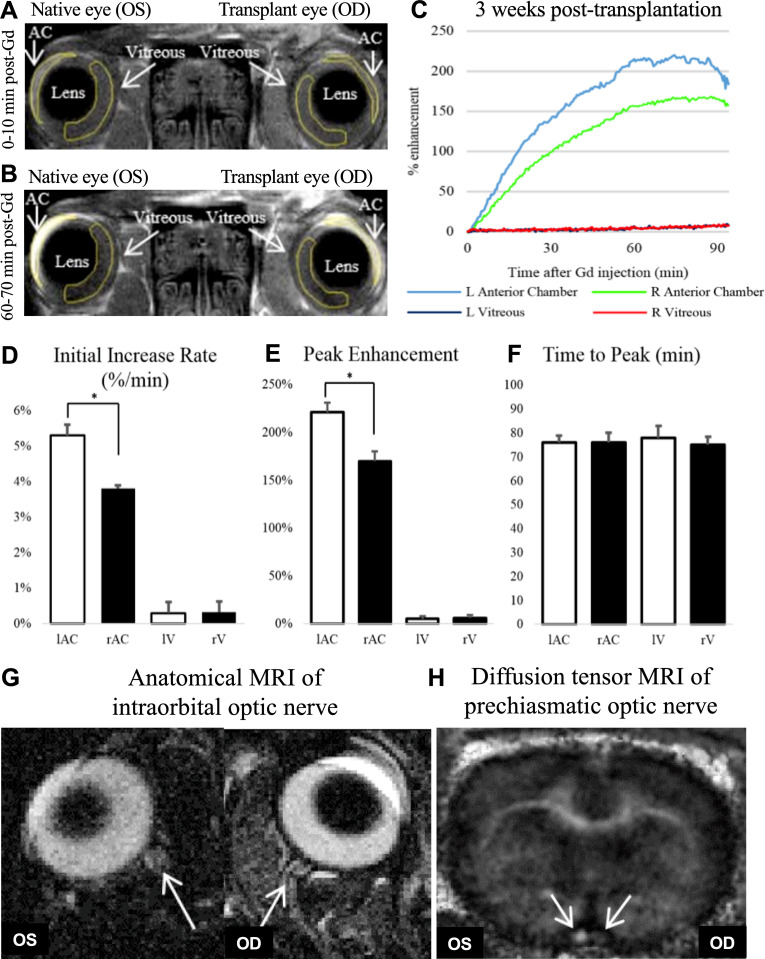
Gadolinium-enhanced imaging (Gd-enhanced MRI) can be used to demonstrate restoration of aqueous humor dynamics after WET. Gd-enhanced MRI of aqueous humor dynamics T1-weighted images at A) 0-10 min and B) 60-70 min after systemic Gd contrast agent administration, showing obvious Gd enhancement in the anterior chamber (AC) but not the vitreous or lens in both eyes at PO week 3. C) Average time courses of % Gd enhancement in anterior chamber and vitreous of both eyes at PO week 3. D-F) Quantitative comparisons of D) initial rate of gadolinium increase E) peak % gadolinium signal enhancement and F) time to peak in the AC and vitreous (V) of native (OS) eye and transplanted (OD) eye at PO week 3 (two-tailed paired t-tests, *P* < 0.01). G) Anatomical T2-weighted images of the intraorbital optic nerves (arrows) projected from the native (OS) eye and transplanted (OD) eye at PO week 3 after WET. Note the presence of the optic nerve sheath surrounding the optic nerves of both eyes. H) Fractional anisotropy map of the prechiasmatic optic nerves (arrows) in the recipient rat using diffusion tensor MRI. Lower fractional anisotropy was observed in the optic nerve projected from the transplanted (OD) eye indicative of compromised white matter integrity.

Transplanted eyes (n = 4) underwent gadolinium-enhanced MRI, anatomical T2-weighted MRI, and diffusion tensor MRI to assess aqueous humor dynamics, ocular tissue permeability, morphology, and white matter integrity of the optic nerve at PO week 3 [37,38]. Warm ischemia time for WET in this cohort was 160.0 ± 18.5 min (mean±SD). Immediately prior to testing, animals were examined and found to be absent of corneal defects. IOP was measured prior to MRI for both native and transplanted eyes. Bilateral IOP measurement values were not found to significantly differ between native and transplanted eyes (15.9 ± 3.1 mmHg *vs* 16.5 ± 3.2 mmHg, respectively; mean±SD; paired t-test, *P = *0.44). Fig 7A and 7B are T1-weighted images of gadolinium enhancement in the anterior chamber of native and transplanted eyes.

Both native and transplanted eyes had similar rates of enhancement at 0-10 minutes post Gd administration and then at 60-70 minutes post Gd administration. The time course of vitreous and anterior chamber enhancement is shown in Fig 7C. There was no difference in the time to peak enhancement in the anterior chamber between native and transplanted eyes, although the transplanted eye exhibited a significantly lower peak intensity and lower initial increase rate than the anterior chamber of the native eye (Fig 7D-F; two-tailed paired t-tests, *P < *0.01). Limited Gd enhancement was observed in the vitreous with no significant difference between native and transplanted eyes (two-tailed paired t-tests, *P > *0.05; Fig 7C-F). Anatomical T2-weighted images showed that the gross morphology of the intraorbital portion of the optic nerve in the transplanted eye was comparable to the corresponding structural morphology of native eyes at PO week 3 (Fig 7G). Diffusion tensor MRI of the prechiasmatic optic nerve showed 54.0 ± 6.1% lower fractional anisotropy, 24.9 ± 5.7% lower axial diffusivity (λ//), and 83 ± 29.5% higher radial diffusivity (λ┴) on the transplanted side compared with the native side (two-tailed paired t-tests, *P < *0.05).

### Electroretinography (ERG)

**Fig 8:**
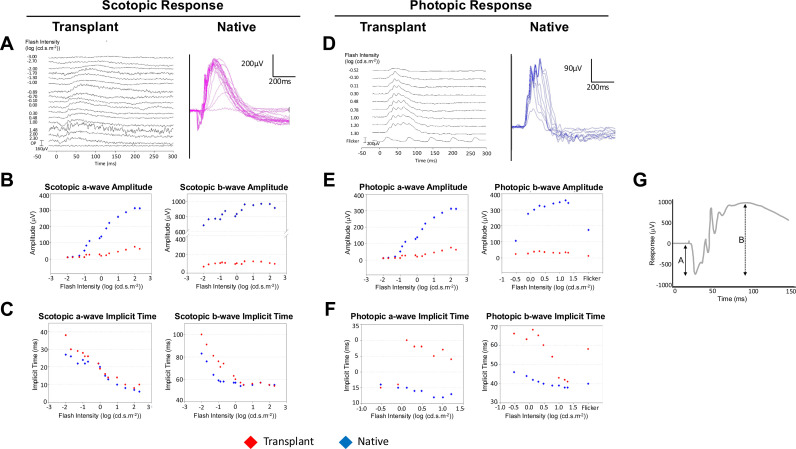
Transplanted eyes exhibit minimal scotopic and photopic ERG responses to stimuli after WET. A) Representative display of all scotopic responses from native and transplant eyes recorded from one dark-adapted rat stimulated with increasing light intensity ranging from 0.0001 – 200.0 cd * s * m^-2^ revealing present but attenuated rod response from photoreceptor of transplanted (OD) eye *vs* contralateral native (OS) eye at PO week 7. B) Representative a-wave and b-wave amplitude and C) implicit time/latency under scotopic conditions for both the native and transplanted eyes. See SF4 for mixed-model analysis of animals (n = 6). D) Photopic (cone-driven) ERG measured from the only animal (1/6) that responded bilaterally to light stimulus ranging from 0.0001 – 16.0 cd * s * m^-2^. E) Summary of a-wave and b-wave amplitude and F) implicit time under photopic conditions for both the native (OS) eye and the transplanted (OD) eye from one of six animals that displayed measurable ERG response to light stimulus ranging from 0.3 – 16 cd * s * m^-2^. During a recording, dark-adapted eyes are exposed to standardized stimuli and the resulting signal is displayed showing the time course of the signal’s amplitude/ voltage. The retinal responses are recorded simultaneously. Signals are measured in microvolts. G) Schematic of a-wave and b-wave measurements under scotopic conditions. A-wave amplitude is measured from the baseline to the first trough (solid arrow). B-wave amplitude is measured from the a-wave trough to the following positive peak (dashed arrow).

At PO 7 weeks, full-field ERG was used to assess the electrical response of transplanted and native eyes (n = 6) under both scotopic and photopic conditions. Warm ischemia time for WET in this cohort was 139 ± 12.5 min (mean±SD). Prior to testing, animals were examined and found to be absent of corneal defects. All the dark-adapted native eyes (6/6) responded under scotopic conditions, whereas five of six (5/6) of the dark-adapted transplant eyes had a very limited response to increasing intensity of light stimuli (Fig 8A). The minimum stimulus to produce a scotopic response was 0.05 cd * s * m^-2^ for both native and transplanted eyes (Fig 8A).

For dark-adapted animals, both the a-wave and b-wave implicit time and a-wave amplitude exhibited clear sigmoidal (logistic) behavior, so sigmoidal mixed-effects models were fitted for data analysis (SF4). Under scotopic conditions, the amplitudes of a-wave and b-wave in the transplanted eyes were consistently lower than those of the native eyes (lower and upper asymptote respectively: *P < *0.0001) and the inflection point occurred at a higher light intensity (*P < *0.0001) whereas the scale factors were similar (*P = *0.26; Fig 8B, SF4). Maximal a-wave amplitude was recorded at a light intensity of 2 cd * s * m^-2^ for both conditions, but the transplanted eye was at least 6-fold lower in amplitude. At the same flash intensity (2 cd * s * m^-2^), scotopic b-wave amplitude for native eyes was approximately 9.5-fold greater than the transplanted eyes (Fig 8B). Transplanted eyes had prolonged scotopic a-wave and b-wave implicit times compared with the native eyes. A bigger effect was observed in transplanted b-wave rather than the transplanted a-wave when compared with native eyes (lower asymptote: *P < *0.0001) and the inflection point occurred at a higher light intensity (*P < *0.0001) whereas the scale factors were similar (*P = *0.26). At the lowest flash intensity, the a- and b-wave implicit time was 2.72-fold and 1.25-fold longer, respectively (SF4). Implicit time decreased in both transplant and native eyes as light intensity increased (Fig 8C, SF4). For transplanted eyes, the decrease in a-wave implicit time with increasing light intensity was not linear like that of native eyes and reduction of b-wave implicit time for both conditions (SF4). Additionally, the rate of decrease in b-wave implicit time (slope) was faster for transplanted eyes compared with native eyes. This effect was reversed for a-wave implicit time (Fig 8C, SF4).

Under photopic conditions, all native eyes (6/6) responded, but only one transplanted eye (1/6) responded to light stimulation (Fig 8D). A-wave amplitude was significantly lower (nearly 6-fold) for the one transplanted eye compared with native eyes when stimulated at a light intensity of 2.0 cd * s * m^-2^ (Fig 8D ). At PO week 7, native eyes had a 13.3-fold higher b-wave amplitude than the transplanted eye at 10.0 cd * s * m^-2^ (Fig 8E). The a-wave implicit time was 2-fold longer in the transplanted eye compared to native eyes (Fig 8F). The photopic b-wave implicit time was initially greater in the transplanted eye *vs* native eyes, but this was reversed when using light stimuli above 10.0 cd * s * m^-2^ and the implicit time was then significantly prolonged in the native eye compared with transplanted eyes (Fig 8F).

## Discussion

This study establishes an orthotopic WET model in rats, demonstrating the technical surgical feasibility of the model and its utility as a foundation for studying the outcomes following WET. We present limited preliminary outcomes using a series of clinical examinations, OCT imaging, electrophysiology, MRI, and histology. Non-invasive methods of testing will be critical for clinical WET. We show these methods can be used to collect data on the health of the eye, assess changes in morphology, and monitor eye physiology and functionality. The 100% surgical survival rate of recipients and long-term health (PO week 7) demonstrate this to be a reliable and reproducible model. Moreover, the aim of maintaining relative morphological integrity of a vascularized composite eye transplant was demonstrated by noninvasive ophthalmological assessments including ocular exam (Fig 4) and OCT scans (Fig 5). OCT findings were confirmed by histology (Fig 6). Overall, all segments of native eyes appeared normal throughout the duration of the study, but transplanted eyes presented mild chemosis of the eye lids, mild ciliary flush of the conjunctiva, cornea neovascularization, mild engorgement of the vessels in the iris, and mild opacities in the lens in some animals. Most of these findings improved over time after transplantation (ST1). Although transplanted eyes were vascularly intact with a patent vascular network, all of their pupils were dilated and unreactive, possibly due to damaged optic and ciliary nerves or anterior segment ischemia [39]. Restoration of vascularity to the VCA eye graft and other physiological and functional assessments were also conducted including tonometry to record IOP (SF3), MRI to assess aqueous humor dynamics (Fig 7), ODT to assess blood flow (SF2, SM1, SM2), and ERG (Fig 8) to assess retinal function after WET.

The number of additional tests that can be conducted on this WET model are too many to list. This apparent study limitation, however, could also be considered as a testament to the utility of our WET model. Future assessment of this WET model should consider evaluating consensual pupillary light reflex to the native eye, which exists in rats (though small) and is independent of oculomotor innervation and could indicate whether the efferent arm of the reflex has been completely restored. Pupillometry is another potential non-invasive, longitudinal measure of optic nerve function. Future studies may take advantage of the modular nature of this WET model and choose to restore the pupil response, blink reflex, and eyelid control as desired to address this issue. Restoration of blink reflex by repairing Cranial Nerves (CN) V and CN VII could reduce exposure and improve the health of the transplanted eye. Furthermore, integrity of the anterior segment tissues including eyelid function and pre-ocular tear film integrity will most likely affect patient comfort, cosmesis, and function, all issues that complicate clinical WET. In addition to the tests conducted herein or previously mentioned, esthesiometer measurements of corneal sensitivity to mechanical stimuli could show the physiology of an expanded version of our model that retains motor output in response to afferent signaling from the trigeminal nerve. Recording visual evoked potentials (VEP) to monitor the integrity of the afferent visual pathway will have a greater role in future studies that incorporate neuroregenerative strategies for the transected optic nerve. Finally, male rats were used exclusively for their physical size, making surgery easier. It has not escaped the authors that future work should consider associated sex differences and inclusion of female rats.

Restoration and maintenance of vascular perfusion to the graft is critical for successful transplantation. We observed perfusion for all WET ocular and facial allografts that presented pink viable skin, indicating restoration of vascular blood supply (Fig 3, SF1). Likewise, ODT scans corroborated the presence of blood flow in the transplanted retina (SF2, SM2). Our model can be used to explore the impact of IRI in the setting of WET and to elucidate IRI mechanisms that remain largely undefined [40,41]. An understanding of these processes will be critical for the success of clinical WET. Future work on the restoration of vascularity should include fluorescein angiography (FA), which allows visualization and measurement of the circulation of the retina and choroid. FA could be used to measure filling of the choroid and retinal vasculature to reveal potential capillary leak, and also record potential changes in fill time among other issues following WET induced by IRI or retinal hypoxia [42]. These events might result in subsequent neovascular glaucoma and chorioretinopathy, respectively [43]. Mainly composed of a blood aqueous barrier and a blood-retinal barrier, the blood-ocular barrier necessitates alternative nourishment and protection of the avascular eye components. FA can be used to assess both the integrity of the vascular system and also the integrity of the blood-ocular barrier.

Aqueous humor is a clear fluid that fills the anterior segment and posterior chambers of the eye, acting in place of blood to support the avascular lens and the cornea. Regulation of the production and drainage of aqueous humor is critical for maintaining physiologically appropriate IOP [44,45]. We recorded IOP in two WET cohorts, for rats that underwent OCT and also prior to MRI recordings. For both cohorts, native eyes were hypotonic compared with either naïve or transplanted eyes at all timepoints, although the significance varied depending on the comparison. Comparing IOP for naïve *vs* transplanted or transplanted *vs* native eyes did not reveal significant differences at all timepoints (S3). Moreover, the mean IOPs recorded for naïve, native and transplanted eyes were similar to the mean IOP value of ~ 17.13 mmHg previously reported for naïve Lewis rats in a comprehensive study conducted by Mermoud *et al.* [46] IOP reading for transplanted eyes was within the reported normal range, although the observations of increased CCT for transplanted eyes were likely secondary to corneal edema. Thicker or steeper CCT is known to elevate measured IOP due to reduced corneal flexibility, and the effect is even greater particularly when presented in conjunction with other corneal pathology [47]. After WET, transplanted eyes indicated ocular surface deterioration as well as corneal and conjunctival pathology. These included: conjunctival hyperemia, chemosis, corneal epithelial defect, infiltration, and neovascularization (ST1, SF2B). These findings could result from a damaged blink response, exposure keratopathy, hypoperfusion following WET, or ischemia of the flap and anterior segment. It’s difficult to determine what to make of our observations, especially as the reported normal mean IOP from Mermoud *et al.* was derived from a very large IOP range, from 7.28-26.98 mmHg. Excepting for cannulation of the eye for IOP accuracy, the transplanted eye IOP measurements indicate some functionality of homeostatic mechanisms and trabecular tissues after WET. Although transplanted eye IOP is within the normal reported range, our Gd-MRI results suggested some compromise in functional homeostatic mechanisms (Fig 7D and 7E) [48].

Monitoring aqueous humor dynamics by Gd-MRI involves systemic administration of exogenous Gd to passively trace aqueous humor flow, which begins with its secretion from the ciliary processes and then circulates to the posterior chamber *via* the blood–aqueous barrier. Contrast agents such as Gd chelates are highly water-soluble, extracellularly distributed, and eliminated rapidly through renal glomerular filtration Such agents serve to improve the sensitivity and specificity of MRI, allowing for easier differentiating between an area of interest and its surroundings [49]. As Gd diffuses and follows aqueous humor flow, it accumulates in the anterior chamber and can be measured by drawing regions of interest (ROIs) on the anterior chamber of both eyes and obtaining their signal time profiles in T1-weighted MRI [50,51] Fig 7C-F show no difference in signal time profiles of the vitreous between transplanted and native eyes at PO week 3. This suggests that the aqueous-vitreous barrier and blood-retinal barrier were preserved in transplanted eyes and that exogenous Gd could not leak into the vitreous. Despite this, we observed a modest but significantly slower initial appearance of the Gd signal in the transplanted eyes when compared with the native eyes. The overall amplitude of the Gd signal in the transplanted eyes was also significantly (*P < *0.01) smaller than that in the native eyes. These observations suggest slower Gd entrance and aqueous inflow to the transplanted eyes. This slowing might arise from the effects of IRI [52]. Therefore, future longitudinal Gd-MRI studies to monitor the remodeling of aqueous humor dynamics in transplanted eyes, and DTI studies should be performed in conjunction with strategies to minimize IRI and restore neuronal structure and function of the visual system using this WET model. It will be interesting to use Gd-MRI to see if any long-term reorganization of the vascular system and potential angiogenesis of small vessels feeding the system might promote recovery over periods greater than PO week 3. Such reorganization occurs in other VCA surgeries such as face transplantation [53]. Other possibilities that might account for a slower aqueous inflow include observed differences in perfusion after transplant, immediately and longitudinally in different tissues. This has been reported in syngeneic rat pancreas-duodenum transplantation, where blood perfusion of the graft is markedly changed whereas islet blood perfusion remains constant [54]. Additional work with this model should seek to provide greater detail of aqueous humor dynamics following WET, at a finer level and include examination of aqueous humor dynamics using other imaging techniques such as FA.

Ischemia is unavoidable in any transplantation, and graft survival is highly dependent on minimizing the potential damage incurred by duration of ischemia and subsequent reperfusion. We presented changes in CCT and TRT following WET using both histology and OCT scans, demonstrating that transplanted CCT increased relative to native or naïve eyes, whereas transplanted eye TRT decreased relative to native or naïve eyes (Figs 5 and 6). These changes could be due to ischemia itself or inflammation secondary to ischemia [55]. Accordingly, we observed anterior chamber fibrinous reaction, which is a sign of ocular ischemia and is associated with corneal epithelial irregularity and thickening of the cornea [56]. TRT and CCT measurements differed when measured from OCT scans or histological preparations (Figs 5 and 6). For example, at PO week 7, transplant CCT measured 247.18 ± 55.47 µm by OCT and 342.34 ± 80.03 µm in histological sections, although in both cases, this thicker CCT represented approximately ~ 2-fold increase relative to native CCT. OCT measurement of CCT for male rats was reported as 159.08 ± 14.99 μm (Min 122.3 μm, Max 181.3 μm). Meanwhile, reported CCT from paraffin slides was 126.89 ± 11.11 μm in eight Lewis rats and the authors described that the fixation process induced shrinkage of up to 20% compared with the living cornea [57]. Aside from potential distortion in measurements from histological processing, the ischemia time for the two cohorts might influence measured values. For the WET histological samples at PO 3 and 7 weeks, mean warm ischemia was 120.0 ± 14.2 min (mean±SD) whereas mean warm ischemia for the WET OCT cohort was 138 ± 16.8 min (mean±SD).

The CCT for naïve eyes measured by OCT averaged 205.67 ± 1.92 µm for the entire study (mean±SD). Previously, CCT for male Brown Norway rats measured by OCT was reported as 159.08 ± 14.99 μm (mean±SD; Min 122.3 μm, Max 181.3 μm; weight: 391 ± 64 g; age: 10–28 months). TRT has been reported to be 201.0 ± 6.00 (μm±SEM) [58] and either 132.0 ± 21.4 (μm±SEM) for 3 months old (considered as young), or 210 ± 31.6 (μm±SEM) for 12 months old (considered as adult) Sprague–Dawley male rats, measured by Scanning electron microscopy [59]. Histological measurement of TRT in 13 week old male Wistar rats weighing ~ 250g, is reported to be ~ 212.5 μm [60]. Accordingly, measurement of CCT and TRT for naïve rats used in this study fell within the previously reported range. Our OCT imaging at PO week 3 showed that the retinas in the transplanted eyes were thinner relative to the native eyes, and that the inner retinal hyperreflective zone was thicker in transplanted eyes, which represents inner ischemic retinal tissue. In native eyes, it represents the nerve fiber layer, and that the choroid is thinner in transplanted eyes. These results were present when retinal layer changes were measured in histological samples (Fig 6). Altogether, the model reveals that IRI remains a major challenge in successful WET. Histological measurements of individual retinal layers of transplanted eyes revealed that inner retinal layers such as the inner plexiform layer (IPL) and retinal ganglion cell layer (GCL) suffered greater relative loss in thickness after WET whereas outer layers such as the outer nuclear layer (ONL) and the photoreceptors (PL) showed some sparing and had greater survival when compared with native or naïve retinal layers at PO week 7 (one-tailed paired t-tests, *P = *0.01).

Corneal and retinal OCT and histology demonstrated persistent changes to the native recipient eye following WET (Figs 5 and 6). Native TRT decreased to 185.8 ± 3.55 µm at PO week 1, representing ~ 85% of naïve TRT. This thinning was stationary in subsequent assessments, measuring 177.4 ± 7.44 µm at PO week 7 (Fig 5D). Changes to cornea and retina were not as dramatic as measured by OCT *vs.* histology, possibly due to the various effects of histologic preparation, such as shrinkage, swelling or dehydration. For example, OCT measurement of retinal thickness at PO week 7 found transplant eyes decreased 50.2% when compared with native eyes, whereas this was markedly higher at 77.8% loss in histological samples. Previous studies have shown good correlation between OCT and histological values; however, researchers should be cautioned to consider the methodologies that are employed [61,62]. Regardless, the loss in TRT after optic nerve transection is not without precedent and our model provides a platform to study potential optic nerve regeneration and RGC preservation in the setting of WET [63,64]. The cause of our observation is unknown and should be explored in future work.

Although this study did not involve neurodegenerative or neuroprotective therapies to address the retinal degeneration typically associated with optic nerve injury, functional testing was performed to establish a baseline and assess the potential for functional recovery. Electroretinography (ERG) is a common non-invasive method to objectively measure functional changes of the retina, by recording the electrical responses of retinal cells to a light stimulus in either dark-adapted (scotopic) or light-adapted (photopic) conditions. A reduction in ERG amplitude is expected after optic nerve injury and this correlates with our histological findings (Fig 6) [65]. Although the gross appearance of the transplanted retinal structure appears intact at PO week 3 (Fig 6), the measurements of TRT of transplanted eyes reveal substantial loss over time. Increased b-wave implicit time could arise from a dysfunction in ON and OFF pathways in the middle retinal layers (primarily from amacrine cells) and rod bipolar pathways [66]. This possibility is supported by our observation of marked recession of those retinal sublayers. In transplanted eyes, the GCL exhibited greater loss than the PL compared with native eyes, in agreement with reports that RGCs and cells of the inner layers are less resistant than the outer retina to ischemic and hypoxic stress. It is known that normal photoreceptor proteins exist in degenerated rods and cones, suggesting that these cells may be capable of functional regeneration [67–69], and display a greater resilience to hypoxia along with ischemia by outer layer retinal cells. This may account for the sparing of albeit weak photoreceptor functionality after WET, despite retinal degeneration that follows optic nerve transection. Future studies to examine these phenomena might include differential labeling of the photoreceptor layer by using rod and cone specific staining, combined with RGC-specific stains.

As functional visual return remains the ultimate goal, this model provides a foundation for future translational strategies and is ideal for testing immunomodulatory, neuroprotective, and neurodegenerative approaches either individually or in combination, as required for total human eye allotransplantation (THEA) to become a clinical reality.

## Conclusions

To date, no other vascularized orthotopic rodent WET transplantation models have been described in the literature. The advantages of an orthotopic model are numerous but the simplest is the obvious anatomic relevance to a future clinical WET procedure. The preference for orthotopic rather than heterotopic models extends to areas of biomedical research, including pancreatic tumor mouse models. Of the two, orthotopic tumor mouse models are preferred because they offer tissue site-specific pathology, allow studies of metastasis, and are generally deemed more clinically relevant. An orthotopic model of WET with repair of the donor and recipient optic nerves also allows for the study of optic nerve regeneration and cortical reintegration into the visual cortex. Importantly, we have developed an orthotopic model that is highly modular with surgical options to allow for additional investigation of other topics directly related to WET [70–72]. Overall, we anticipated many of the transplant pathologies observed in this novel WET model. We have demonstrated its potential use to study axonal regeneration beyond the optic nerve to include other CNS trauma and disease or complications that follow VCA.

### Methods

#### Animals.

Syngeneic transplantations were performed using 23 age-matched 10 to13 week-old pairs of male Lewis (RT1) rats (Charles River Laboratories, Inc., Wilmington, MA) with weights ranging from 290g-350g. An additional 8 animals were used in the event that ocular damage precluded subsequent measurements with OCT and ERG (Fig 1). These additional WET surgeries were created to maintain appropriate sample size per group per time and were not included in the ocular examination under anesthesia and histologic analyses. Six naïve animals were used for strain, sex, and age-matched controls. All animals received care in compliance with the University of Pittsburgh Institutional Animal Care and Use Committee, following the guidelines from the Guide for the Care and Use of Laboratory Animals published by the National Institutes of Health. The University of Pittsburgh provided veterinary care in a pathogen-free animal facility. The animals were provided with nutrition and water *ad libitum* and caged in groups of three with a 12-hour light/dark cycle prior to surgery and in pairs post-transplantation.

#### Surgical care.

All surgeries were performed by clinical surgeons with micro-vascular training, working as a team of two, using a dual headed Zeiss Superlux 300 Surgical Microscope (Carl Zeiss Microscopy, LLC White Plains, NY). Procedures were done under sterile conditions, with both donor and recipient animals kept on a heating pad (Braintree Scientific, Inc., Braintree, MA) for maintaining body temperature of 37°C. Induction of anesthesia was performed with 80 mg/kg of ketamine/xylazine, administered intraperitoneally. Anesthesia was maintained with 40 mg/kg/h of ketamine/xylazine (IP) when necessitated by pedal response. Glycopyrrolate (0.5 mg/kg) and dexamethasone (4 mg/kg) were administered intramuscularly immediately after initial induction of therapy to reduce secretions and airway congestion. Qualitative monitoring was used to monitor normal cardiovascular and respiratory function/effort during surgery, as indicated by pink and moist mucous membranes and capillary refill time that was less than 2 seconds.

#### Operative technique.

Preparation of WET Donor. An elliptical incision was made around the eye and the auricle, leaving a 0.5-cm margin of skin. A linear incision was then made continuously with the elliptical incision to expose and dissect the common carotid artery and external jugular vein. After isolating the vascular pedicles, the dissection continued superiorly in a subperiosteal plane overlying the superior and anterior portion of the cranium. Flap elevation continued anterior to the masseter muscle. The orbital contents were isolated from the osseous orbit and raised with the flap. The zygomatic arch and surrounding muscles were removed. The muscles of the neck were severed from the attachments to the cranial bones. The external jugular vein and its branches were isolated at the proximal end. The common carotid artery was exposed, after cutting the posterior belly of the digastric muscle. The internal carotid system was procured and elevated with the flap. The branches of the external carotid artery, with the exception of the maxillary artery and posterior auricular artery, were ligated. The flap, while remaining attached to the rat, was perfused with heparinized Lactated Ringer’s solution after sacrificing the rat with carbon dioxide. The optic nerve was transected at the chiasm. The flap was stored at 4 ⁰C awaiting transplantation (Fig 2).

Preparation of WET Recipient. The skin and the soft tissue overlying the masseter, the temporalis, and the sternocleidomastoid muscle ipsilateral to the donor flap were removed in the recipient rats. The facial nerve was kept intact. A segment of the temporalis muscle above the zygomatic arch was removed to facilitate the coaptation of the optic nerve and to provide space for the donor tissues. Exenteration was performed by removing the orbital contents with the optic nerve transected at the base of the globe. The external jugular vein was isolated and prepared for venous anastomosis. The sternocleidomastoid muscle was retracted to expose the common carotid artery for anastomosis (Fig 3).

#### Transplantation and flap insetting.

The flap was secured to the recipient with three interrupted 5-0 chromic sutures. Microvascular anastomoses and nerve coaptations were performed using an operating microscope. Arterial anastomosis of the common carotid artery of the donor and the recipient was performed using standard end-to-end microsurgical technique with 11-0 nylon sutures. The donor and the recipient external jugular veins were anastomosed using a cuff technique with 9-0 nylon sutures. Optic nerve apposition was performed with interrupted 11-0 epineural nylon sutures, with an effort to preserve globe orientation and minimize optic nerve twist. Inset was completed with interrupted 5-0 chromic sutures. The eyelids were closed with a blepharorrhaphy suture using 9-0 nylon sutures (Fig 3).

#### Postoperative care.

Rats were given Transgel (Charles River Laboratories, Inc., Wilmington, MA) and a soft-pellet high-fat diet (BioServ, Flemington, NJ) to ease oral consumption and to maintain proper weight gain in the postoperative period. For postoperative analgesia, all transplant recipients received ketoprofen (5mg/kg) subcutaneously every 24 hours for 72 hours. A 5mL bolus of Lactated Ringer’s solution was intraperitoneally administered immediately after donor flap transplantation, followed by once per day for 3 days for postoperative hydration. A tarsorrhaphy was performed for corneal protection immediately after WET surgery. Tarsorrhaphy was removed prior to EUA, OCT, ERG, and MRI then immediately replaced. Recipients were weighed daily until their preoperative weight was recovered and their general health was monitored throughout the postoperative period.

#### Ocular examination under anesthesia (EUA).

Prior to ocular exam, rats were anesthetized with 80mg/kg of ketamine/xylazine by intraperitoneal injection. The external and anterior segment exam were performed using an ophthalmic operating microscope. The integrity and perfusion of the periorbital flap tissue and eyelids were evaluated on external exam. Conjunctival, corneal, anterior chamber, iris, pupil, and lens findings were assessed and described, based on human ophthalmic standards, whenever there was adequate view for this evaluation. The retina and vitreous were examined using indirect ophthalmoscopy with 78-diopter lens following coating of the ocular surface with hydroxypropyl methylcellulose (Goniosol). Retinal perfusion was reported based on visible perfused vessels both in central and peripheral retina. Central retina was defined as the area with radius of 2 optic disc diameters from the optic nerve.

#### Optical coherence tomography (OCT).

After general anesthesia was induced by an intraperitoneal injection of 80 mg/kg of ketamine/xylazine, the pupil was dilated with 1 drop each of 0.5% Tropicamide (Alcon, Fort Worth, TX) and Goniovisc 2.5% (Hypromellose, HUB Pharmaceuticals, LLC., Scottsdale, AZ) to prevent corneal desiccation. A spectral-domain OCT (Bioptigen, Durham, NC) equipped with a wide-bandwidth light source centered at 870 nm (Superlum, Dublin, Ireland) was used to visualize a 2.5 mm x 2.5 mm x 2 mm sample of the cornea, lens, and optic nerve head (ONH) with 512 x 512 x 1024-pixel samplings. For retinal OCT, rats were positioned such that the fundus was clear, and the optic disc centered. Each subject was scanned 1-3 times as necessary to produce a single scan/subject for analysis. Doppler OCT was performed in a 2.5mm x 2.5mm x 2mm (700 x 20 x 1024 samplings) region. Eight Doppler frames were acquired with an acquisition rate of 28 kHz, resulting in a Nyquist limit of 4.62 mm/s normal to the scanning beam.

Morphometric measurements were restricted to retinal regions outside a radius of 500 mm from the center of the ONH. Retinal layer measurements were performed via the automated segmentation software provided by the instrument manufacturer (Bioptigen, Inc., Durham, NC). Corneal OCT measurement of corneal thickness was performed using Metamorph software, of the central part of the cornea from the epithelium to the endothelium. For OCT measurement of the retina, the central image of a cube OCT scan centered over the optic nerve head containing the widest diameter of the central vessels was chosen and imported into Metamorph (version 4.5r) Professional Image Analysis software, produced by Universal imaging, USA (ImagxCell). Then, from the centermost part of the vessel, 2 lines parallel to the inner retinal surface, each with a length of 100 pixels, was drawn outward on the two sides of the optic nerve. The following measurements were performed at 100 pixels from the center of the optic disc and perpendicular to the retina. TRT was measured from inner retina to the outer hyperreflective band. The inner hyperreflective zone (most probably representing nerve fiber layer in normal eye, and nerve fiber layer plus ischemic retina in transplanted eye) was measured from the inner retinal surface to the outer boundary of the inner hyperreflective layer. All measurements were then exported to Excel for analysis by Metamorph. A conversion factor of 1.6 for retinal images was used to change pixel measurements into micrometers (as the depth of OCT measurements was 1.6 mm for 1024 image pixels in the retina OCTs). For each of the thicknesses, the mean of the two corresponding measurements on the two sides of the optic disc was used as the representative value for each eye.

#### Gadolinium-enhanced magnetic resonance imaging (Gd-MRI), anatomical T2-weighted MRI and diffusion tensor MRI.

A subset of WET recipients (n = 4) underwent Gd-MRI, anatomical T2-weighted MRI, and diffusion tensor MRI at PO 3 week to assess aqueous humor dynamics, ocular tissue permeability, and the morphology and white-matter integrity of the optic nerve. At PO 1 week and 3 weeks, before MRI experiments, IOP was measured bilaterally according to manufacturer directions using the handheld Toolbar rebound tonometer (Icare, Finland) within 5 min after isoflurane gas anesthesia induction in the afternoon. Additionally, IOP of age-matched naive animals was measured bilaterally for the same time points. Measurement for each eye was repeated 3 times and the average IOP and deviation was determined for each group (naïve, native, transplant). MRI scans were performed using a 9.4-Tesla/31-cm Varian/Agilent scanner with a transmit-receive volume coil for Gd-MRI and T2-weighted MRI, and with a volume transmit and surface receive coil for diffusion tensor MRI. For Gd-MRI, gadolinium-diethylenetriamine penta-acetic acid (Gd-DTPA, Magnevist), 0.3mmol/kg, was introduced intraperitoneally after obtaining a baseline T1-weighted image. T1-weighted imaging at 30s-temporal resolution was then continuously acquired for 1.5 hours using a fast spin-echo sequence with the following imaging parameters: repetition time/echo time = 600/8ms, echo train length = 8, in-plane resolution = 135x135µm^2^, and slice thickness = 1mm. Slices were oriented to bisect the center of the eyes. Anatomical T2-weighted MRI was acquired using a standard spin-echo imaging sequence. Diffusion tensor MRI was acquired before Gd-MRI using a fast spin-echo sequence with 12 diffusion gradient directions at diffusion weighting factor (b) = 1.0ms/μm^2^ and two b = 0ms/μm^2^. Other imaging parameters included: repetition time/echo time = 2300/27.8ms, echo train length = 8, duration of diffusion gradient pulses (δ)/time between diffusion gradient pulses (Δ) = 5/17ms, acquisition resolution = 135x135µm^2^, and slice thickness = 0.5mm. Slices were oriented orthogonal to the prechiasmatic optic nerves. (FA) axial diffusivity (λ//) and radial diffusivity (λ┴) were computed using DTIStudio. ROIs were drawn on prechiasmatic optic nerves at approximately 3.5mm rostral to Bregma and compared bilaterally.

#### Histologic evaluation and analysis.

Prior to animal sacrifice at PO 3- and 7- weeks, eye globes were enucleated and immediately fixed in 4% paraformaldehyde. After 24-36 hours, eyes were transferred into 70% ethanol and stored for subsequent embedding in paraffin. Blocks were sectioned (10µm) and every twentieth section was stained with hematoxylin & eosin (H&E). Sections were independently evaluated by two trained individuals for all specimens in the study using digital images acquired from histological slides. Using ImageJ [73], the retinal sub-layers and total thickness of retinal sections were measured, and the values reported as an average of measurements obtained by the independent investigators as previously described [74]. Briefly, for each eye, one histological section (the middle most) was imaged using a Nikon Eclipse 80i microscope (Nikon USA, Melville, NY, USA) with brightfield setting at 20x magnification. A scale bar was inserted in each image using the Nikon NIS Elements software. For total retinal thickness, 3 fields representing naso, central and temporal areas of each section were delineated, then measured at the section midpoint before the three values were averaged. ImageJ was used to set scale and then draw four measurement segments from top to bottom of each retinal layer or central cornea area; segment values were recorded and then averaged to determine the thickness in microns. Retinal layers were analyzed from the same images used to determine total retinal thickness. The central most corneal section per eye was selected for analysis. Final values represent the pooled mean ±  s.d. of the measurements obtained by the two independent researchers, with all values comporting to within 5% between independently acquired data sets.

#### Electroretinography (ERG).

In scotopic ERG, low-intensity light flashes can induce rod activation, so the function of rod photoreceptor and downstream retinal cells can be examined. In photopic ERG, high-intensity light flashes are presented after a period of light adaptation. Under these conditions, there is high cone activation, and the rod response is suppressed. The a-wave on the ERG reflects the responses of photoreceptors and the b-wave originates from the activity of retinal cells downstream from photoreception, mainly bipolar and Müller cells [75].

After overnight dark adaptation (minimum 12 hours), a subset of rats (n = 5) at PO week 7 were anesthetized by intraperitoneal injection of 80 mg/kg of ketamine/xylazine, and pupils were dilated using topical application of 0.5% Tropicamide eye drops under dim red illumination. Full-field ERG responses were recorded using gold-wire loop electrodes precoated with a 2.5% Hypromellose lubricant solution (Goniovisc, HUB Pharmaceuticals). The reference needle electrode was inserted under the skin between the ears, and the ground needle electrode was inserted subcutaneously into the left hind limb. Full-field ERG was performed using an Espion Diagnosys system (Diagnosys LLC, Littleton, MA). ERG data were collected and analyzed with a custom script for MATLAB 8.5 (The MathWorks, Inc., Natick, MA). Only signals greater than three s.d. over noise were included for curve fitting in the analysis of a- and b-wave amplitude and implicit time for both native and transplanted eyes. During recordings, body temperature was maintained at 37°C with a platform heater. Scotopic light stimuli ranged from 0.1x10^-6^ to 200 cd·s·m^ − 2^ using a ColorDome unit to produce a single flash in a light-sealed room. To ensure complete recovery of b-wave amplitude, the interstimulus interval between the six intensities progressively increased from 10 to 120 seconds. After scotopic ERG evaluation, animals were given 10 minutes of light adaptation. The rats then were exposed to photopic light flash ranging from 3x10^-3^ to 16 cd·s·m^ − 2^ ramping over eight intensities in semi-log unit increments. Each record is an average of 20 responses obtained with a 2 second interstimulus interval.

#### Statistical analysis.

Power analysis was used to determine the number of experimental animals used in each group/timepoint/assessment as previously described. Using G * power software (G * power software 3.1.9.2, Germany), we calculated the effect size to be 0.6 according to our previous experiments. Power analysis for ANOVA required n = 4 animals for histological analysis, whereas n = 5 animals is necessary for *in vivo* studies to achieve 80% power for α =  0.05.

Data obtained from MRI analysis of the aqueous humor dynamics were analyzed using a two- tailed paired t-test. Data are presented as mean + /- standard deviation unless otherwise specified. Results were considered significant when *P* <  0.05. STATA or IBM SPSS Statistics for Windows, version 19 (IBM Corp., Armonk, NY) was used to perform the analysis.

Statistical analysis of ERG data was performed using R environment for statistical computing (R version 3.3.2 (2016-10-31, nlme 3.1.129, lattice 0.20.34). After log transformation of light intensities and ERG responses, a well-defined nonlinear pattern was revealed. Log b-wave implicit time responses were sigmoidal, so we used two types of nonlinear mixed effect models for a-wave amplitude and implicit time, and b-wave implicit time. The data were fitted to a sigmoidal model with four parameters: upper and lower asymptotes, inflection point, and scale factor. For the b-wave amplitude, we used an asymptotic model with 3 parameters: asymptote, log of the rate constant, and light intensity offset. 4 parameter logistic function was used for the b-wave log implicit time as a function of log light intensity with parameters:1) A- asymptote as light intensity goes to infinity, 2) B- asymptote as light intensity goes to infinity, 3) Xmid- inflection point for light intensity, and 4) S- scale = difference between Xmid and light intensity where response is 75% of distance from A to B asymptotes. Larger S = shallower slope. Fixed effects were A, B, Xmid, and S for the left/naive eye and their differences with right eyes. Random effects (RE) were included for rats for Xmid (rat) and for eyes for B (eye). Fixed effects (FE) describe typical effect while REs describes rat/eye-specific effects.

## Supporting information

Supporting Fig 1 (SF1). Reperfusion of eye during WET and fundoscopic examination at PO 1 week. A) The pre-op native eye. Vasculature can be seen through the iris. B) In the ischemic phase, the donor eye appears white with opacity in the lens after harvesting and flushing with heparinized saline. C) 1 hour after reperfusion there is hyperemia, and engorgement of the vessels is seen. D) Gross appearance of transplant eye at PO 1 week, appears comparable to the native eye with the exception of corneal neovascularization and a dilated pupil.
(TIFF)

Supporting Fig 2 (SF2). Optical coherence tomography (OCT) at PO 1, 3, 7 of n = 1 transplanted eye reveals A) some formation of lenticular hyper-reflectivity, likely opacities resulting from ischemia, ischemia reperfusion injury or exposure. B) Evidence of neovascularization of cornea of transplanted eye at PO 1 week. A-B) Images are from one animal in the EUA and OCT (n = 6) cohort, Open arrows indicate A) hyper-reflectivity B) neovascularization. C) Fundus images of a. Native and d. Transplant OCT used for b. and e. Enface b-scan (cross sectional views) of the retina, the optic nerve head and the surrounding blood vessels demonstrate the general maintenance of structural integrity of the retina after transplantation. Cross-sectional image was performed at the location of the red line in the enface image. C) c. and f. Doppler Optical Coherence Tomography (DOT) of the retina: Blood flow is demonstrated in the retina of both native and transplanted eyes (blue and red coloring at indicated red arrows are individual vessels indicate bidirectional blood flow).
(TIFF)

Supporting Fig 3 (SF3). IOP measurements. IOP was measured serially at PO 1, 3 and 7 weeks for both native and transplanted eyes of the subset of WET animals used in the OCT cohort (n = 7) and compared with age-matched naïve eyes (n = 6, 4, 2/timepoint respectively). Within each group of eyes (naïve, native and transplant), pooled mean values of IOP were not found to be significantly different over time (15.5 ± 2.1 mmHg, 9.5 ± 3.0, 12.8 ± 2.6 mmHg respectively (mean ±  standard deviation); ANOVA, *P* = 0.8 within each group).
(TIFF)

Supporting Fig 4 (SF4). Dark-adapted ERG 3 week after transplantation showed an organized response to electrical stimulus, whereas the retina in the transplants showed little to no response. C) The mixed-effects model fits for scotopic response in native eyes and transplanted eyes. The sigmoidal curve had four parameters: lower asymptote, upper asymptote, inflection mid-point, and scale factor (slope). The a-wave amplitude showed only part of a sigmoidal pattern and therefore an asymptotic mixed effects model was used. The asymptotic curve had three parameters: upper asymptote, offset, and logarithm of the rate constant.
(TIFF)

Supporting Table 1 (ST1). Summary table of all EUA finding for native and transplanted eyes. Condition of the lid, conjunctiva, cornea, lens, anterior chamber, vitreous, and iris was noted during examinations PO 1-, 3-, 5- and 7- week.
(TIFF)
